# Intensive community care services for children and young people in psychiatric crisis: an expert opinion

**DOI:** 10.1186/s12916-023-02986-5

**Published:** 2023-08-10

**Authors:** Eleanor Keiller, Saba Masood, Ben Hoi-ching Wong, Cerian Avent, Kofi Bediako, Rebecca Margaret Bird, Isabel Boege, Marta Casanovas, Veronika Beatrice Dobler, Maya James, Jane Kiernan, Maria Martinez-Herves, Thinh Vinh Thanh Ngo, Ana Pascual-Sanchez, Izabela Pilecka, Paul L Plener, Karin Prillinger, Isabelle Sabbah Lim, Tania Saour, Nidhita Singh, Eirini Skouta, Mariana Steffen, Jovanka Tolmac, Hemma Velani, Ruth Woolhouse, Toby Zundel, Dennis Ougrin

**Affiliations:** 1 Queen Mary University of London, London, UK; 2 East London NHS Foundation Trust, London, UK; 3 Central and North West London NHS Foundation Trust, London, UK; 4 West London NHS Trust, Southall, UK; 5 Medical University Graz & ZfP Südwürttemberg, Graz, Austria; 6 Sant Joan de Deu Paediatric Hospital, Barcelona, Spain; 7 Cambridgeshire and Peterborough Foundation Trust, Cambridgeshire, UK; 8 South London and Maudsley NHS Foundation Trust, Beckenham, UK; 9 King’s College London, London, UK; 10 Medical University of Vienna, Vienna, Austria; 11 GHU Paris psychiatrie et Neurosciences, Paris, France; 12 East London NHS Foundation Trust & Queen Mary University of London, London, UK

**Keywords:** Children, Young people, Mental health, Community care, Intensive community care services, Inpatient care, Treatment model

## Abstract

**Background:**

Children and young people’s (CYP) mental health is worsening, and an increasing number are seeking psychiatric and mental health care. Whilst many CYPs with low-to-medium levels of psychiatric distress can be treated in outpatient services, CYPs in crisis often require inpatient hospital treatment. Although necessary in many cases, inpatient care can be distressing for CYPs and their families. Amongst other things, inpatient stays often isolate CYPs from their support networks and disrupt their education. In response to such limitations, and in order to effectively support CYPs with complex mental health needs, intensive community-based treatment models, which are known in this paper as intensive community care services (ICCS), have been developed. Although ICCS have been developed in a number of settings, there is, at present, little to no consensus of what ICCS entails.

**Methods:**

A group of child and adolescent mental health clinicians, researchers and academics convened in London in January 2023. They met to discuss and agree upon the minimum requirements of ICCS. The discussion was semi-structured and used the Dartmouth Assertive Community Treatment Fidelity Scale as a framework. Following the meeting, the agreed features of ICCS, as described in this paper, were written up.

**Results:**

ICCS was defined as a service which provides treatment primarily *outside* of hospital in community settings such as the school or home. Alongside this, ICCS should provide at least some out-of-hours support, and a minimum of 90% of CYPs should be supported at least twice per week. The maximum caseload should be approximately 5 clients per full time equivalent (FTE), and the minimum number of staff for an ICCS team should be 4 FTE. The group also confirmed the importance of supporting CYPs engagement with their communities and the need to remain flexible in treatment provision. Finally, the importance of robust evaluation utilising tools including the Children’s Global Assessment Scale were agreed.

**Conclusions:**

This paper presents the agreed minimum requirements of intensive community-based psychiatric care. Using the parameters laid out herein, clinicians, academics, and related colleagues working in ICCS should seek to further develop the evidence base for this treatment model.

## Background

The COVID-19 pandemic prompted the worsening of mental health symptoms, namely anxiety and depression, of children and young people (CYPs) around the world [1]. Following, and in response to such worsening, record numbers of CYPs are now seeking psychiatric and mental health care [2]. Between 2019 and 2020, 4038 CYPs were admitted to inpatient mental health care in the UK’s National Health Service (NHS) [3]. Similarly, in Germany, although a larger overall population than the UK, 62,224 CYPs were admitted in 2021 [4]; this figure increased from 54,626 the previous year [5]. Whilst necessary in many cases, inpatient psychiatric care can be particularly distressing for CYPs and their families (CYPFs) or other support networks [6–8]. The nature of such care means that CYPs are removed from their ‘lives as usual’, they can become isolated from their family and friends and are frequently unable to attend school or social activities. In addition, inpatient psychiatric beds for CYPs are a consistently limited resource [9], and many CYPs are moved far away from home in order to receive appropriate treatment [3, 10]. Emergency psychiatric care is also expensive [11], and the demand on health service resources is particularly high [8]. Long inpatient stays may also be associated with an increase in self-harming behaviours [12] and, upon discharge from inpatient care, CYPs are at a particularly high risk for suicide or self-harm [13, 14]. Finally, it should be noted that inpatient treatment is often limited in terms of addressing the underpinning biopsychosocial factors which contribute to the CYPs initial presentation or mental health crisis. In response to the above limitations, and in order to provide mental health care that can support CYPs with complex clinical needs outside of hospital, organisational models which provide an alternative to inpatient care have been developed. Whilst the existing literature on such organisational models is limited, clinical promise has been identified [15] for treatment delivered in this way. Supported discharge [8, 16], home treatment [17], intensive case management [18], assertive community treatment [19–21] and multisystemic therapy [22–24] are some examples of such models that have shown excellent clinical potential. In this paper, intensive community care services (ICCS) are used to encompass all such services.

ICCS were generally considered to have a number of advantages when compared to inpatient services as described above. ICCS models are often community-centred and treatment takes place in locations preferred by the CYP or family such as the home [25], school, parks and cafés. Such settings prevent the isolation of CYPs from their families and negate the need for complete removal from school and social activities. Community-based settings such as these also support clinicians in the holistic assessment of CYPs circumstances and the subsequent adaption of treatment plans to each context; this, in turn, increases the likelihood of sustainable change and overall treatment benefit. ICCS enables clinicians to work directly with CYP’s families and seeks to reintegrate CYPs into their communities at an early stage following a mental health crisis [25]. ICCS could also prevent CYPs from requiring inpatient psychiatric admissions wherein their primary surroundings are other CYPs in crisis; as such, remaining in the community prevents the vicarious learning of maladaptive behaviours [26] that can take place in inpatient settings. Existing research into models such as ICCS is promising. Recent research has determined that intensive community-based treatments are associated with shorter [10, 25] and fewer [8, 25, 27] hospital admissions, greater patient satisfaction [10, 28], reduced severity of psychiatric symptoms [25, 27, 29] and improved general functioning [25].

The World Health Organization [30] describes community-based mental health care as person-centred, rights-based and recovery oriented, and the benefits of such care, over inpatient care, have not gone unrecognised. A commitment to community mental health services is made in the NHS Long Term Plan [31]. The plan makes particular reference to the treatment of CYPs and commits to embedding mental health care in community settings such as schools and colleges. Community-based psychiatric care is also recommended by the UK-based National Institute for Health and Care Excellence (NICE); community-based care is recommended, in particular, for CYPs with psychosis and schizophrenia [21]. Moves towards ICCS are developing elsewhere also. In Germany, the introduction of insurance which covers ICCS (“Stationsäquivalente Behandlung”) was legally introduced in 2017 making such treatment an accessible alternative to inpatient care [32].

Whilst the move towards intensive community-based psychiatric care is very welcome, there is a need to define the scope and role of intensive community-based services such as ICCS. At present, ICCS and related teams operate in isolation from one another, and there is very little shared or best practice. As it is now, intensive community-based mental health teams are given a variety of names, have different structures and offer differing levels of support to CYPFs [33]. Such service heterogeneity does not only engender a varied level of care for CYPFs, there is also a need to standardise ICCS in order to provide the necessary guidance and policy for health providers and related insurance companies and to integrate ICCS within the existing healthcare landscape [33, 34]. In order to provide effective and fair intensive community-based mental health services, the scope and nature of such services must be agreed and defined. Whilst useful, consensus-based guidelines, such as those modelled by Young et al. [35], are yet to be conducted for ICCS. As such, this paper seeks consensus amongst a group of experts in order to clearly define the remit, boundaries and the minimum level of care for ICCS; doing so may lead to higher-quality, standardised care for CYPFs who turn to such services in times of great need and distress.

This paper presents the findings of a meeting and discussion which took place in January 2023. A small group of experienced clinicians, academics and researchers met to debate and agree upon various factors relating to ICCS. The findings presented herein are the minimum requirements, as agreed by the group, that services should aim to adhere to in order to qualify as ICCS.

## Methods

A panel of nineteen experts, whose work relates to child and adolescent mental health, convened in London in January 2023. The small group, who were from the UK, Austria, France, Germany and Spain, was made up of experienced clinicians (including psychiatrists, psychologists, nurses and allied health professionals) as well as researchers and academics from across the field of child and adolescent mental health. The group members were identified largely via their pre-existing role in a national randomised controlled trial named IVY: Comparison of Effectiveness and Cost-Effectiveness of Intensive Community Care Services versus Usual Inpatient Care for Young People with Psychiatric Emergencies (IVY): An Internal Pilot followed by a Randomised Controlled Trial Comprising All Intensive Community Service Care Teams in Great Britain. The IVY study (ISRCTN: ISRCTN42999542) seeks to evaluate the effectiveness and cost-effectiveness of ICCS compared to usual care (including, but not limited to, inpatient care) in young people with severe psychiatric disorders.

The purpose of the meeting described in this paper was to generate discussion and agreement regarding the nature and scope of ICCS for child and adolescent psychiatry and mental health. The meeting was semi-structured and used the Dartmouth Assertive Community Treatment Fidelity Scale (DACTS) [36] as a framework for the discussion. DACTS is an established fidelity measure for an ICCS model in the US. DACTS guided the discussion through aspects of service provision such as organisational boundaries, human resources, and nature of services, all of which are considered in this paper. For each point, group members were encouraged to share their thoughts and clinical experiences before consensus was agreed by discussion. Most often, unanimous consensus was reached in this way; however, where disagreements arose, further discussion led to either consensus or to the corresponding aspect of ICCS being excluded from this paper. As the field progresses, we, or others, may return to these aspects for further discussion and agreement. Notes were taken by EK and SM throughout the meeting which were displayed live to all group members in order to facilitate collaboration and transparency within the creation of the manuscript.

The above method was selected in order to facilitate an open and free-flowing group discussion on the topic in question. Throughout the discussion, which took place in-person and in real-time, both concurring and divergent views on each topic area were sought and consensus was reached via group decision-making.

Following the discussion, the agreed features of ICCS, as described in this paper, were written into a single document. The document was then circulated to group members and invitees who were unable to attend the in-person meeting and comments were welcomed. At this stage, the document was also shared with a former service user who had been identified as an expert by experience, having received ICCS support when they were a young person (< 18 years). The individual, who was an adult at the time of study, shared valuable insights on the discussion and the subsequent creation of guidelines. The individual also contributed to the critical revision of the manuscript and consented to be named as a co-author of this paper.

## Ethical approval

Following an enquiry to the NHS “Do I need NHS REC review?” decision tool (https://www.hra-decisiontools.org.uk/ethics/), ethical approval was deemed not needed for this conduct of this study. Ethical approval was deemed not necessary as this study was born out of a meeting of professionals and with a youth former service user (now adult) with lived experience. The ex-service user was already known to the authors and was not recruited via NHS services. In other studies, this process has been confirmed by Health Research Association’s Quality and Performance Manager to not require ethical approval.

## Results

The findings presented herein are the minimum requirements, as suggested by the expert group, for ICCS and related services of a different name. A summary is presented in Table 1.

**Table 1:** Summary of the minimum requirements of ICCS as defined by the expert group

Definition of ICCS		Psychiatric treatment is provided, at a high frequency, primarily outside of hospital.
Organisational boundaries	Admission and intake (ICCS)	CYPs admitted to ICCS should comply with explicit pre-determined admission criteria (such as age, severity of need etc).
	Areas of provision	Amongst other things, support may include psychiatric care, psychological therapies, housing, educational, employment and rehabilitative support.
	Hours of support	ICCS should provide, at least, some provision beyond Monday to Friday 9am–5pm.
	As-needed inpatient care	ICCS should aim for involvement in inpatient admission and discharge decisions in collaboration with CYPFs.
	Discharge (ICCS)	ICCS is time-limited and should form a treatment pathway. ICCS should regularly review the needs of CYPFs and check their eligibility for the service.
Human resources	Caseload	Caseloads should be small enough to allow for intensive community work which proffers flexibility and for CYPFs to be seen as, and where, they need.
	Clinician contact	90% or more of the CYPFs on caseload should have a minimum of two episodes of direct clinical contact with at least one clinician each week.
	Team contact	ICCS should meet to discuss all CYPFs at least once per week. The discussion may be brief or detailed depending on each CYPFs need.
	Team lead role	ICCS Team Leads should maintain connection to, and knowledge of, the caseload as a whole.
	Access to MDT	ICCS should have access to at least one professional who is legally able to diagnose, prescribe medication and apply mental health legislation.
	Supervision	ICCS should aim to provide individual clinical supervision at least once per month. A separate space for reflective practice is also recommended.
	Size	The minimum size of a team should be at least four FTE staff members.
Nature and scope of services	Setting	A minimum of 80% of face-to-face contacts for ICCS teams should take place in the community.
	Community engagement	ICCS team members should promote and actively support CYPs engagement with community resources, such as sport and activity clubs.
	Frequency of engagement	ICCS teams should take a considered, flexible and persistent approach to attempting to engage with CYPs. Consent is critical in this approach.
	Method of engagement	ICCS teams should utilise a range of engagement mechanisms and should have a well-considered engagement strategy in place to enable this.
	Frequency of contact	At least 2 hours of direct clinical contact should be provided to each CYPF on the caseload per week.
	Nature of contact	ICCS should meet with CYPs wider network (including family, teachers, community leaders), with or without the CYP present, on a regular basis.
Evaluation		ICCS should consistently monitor CYPs progress using a variety of outcome measures related to mental health and psychological wellbeing.

### Definitions

The expert group determined that the defining feature of ICCS is the nature of the service to provide treatment primarily *outside* of hospital in community settings such as schools, homes, religious/cultural centres and cafés. In addition, the group agreed that in order to qualify as an ICCS, this community-based treatment should be provided at a higher frequency and intensity than regular community treatment teams and should have an in-built adaptability which is not necessarily present in other services. It was noted by the group, however, that within this description there are a vast number of titles currently given to services which constitute ICCS. As such, the following titles were thought to be relevant to ICCS and the preceding discussion in this paper:

- Assertive Community Teams
- Crisis and Resolution Teams
- Assertive Outreach Teams
- Mobile Treatment Teams
- Home Treatment Teams
- Crisis & Home Treatment Teams
- Adolescent Outreach Teams
- Alternative to Hospitalisation/Treatment
- Day Services (which could be ICCS or part of usual community services)
- Intensive Outpatient Services
- Stationsäquivalente Behandlung (Equivalent to Inpatient Treatment)
- Discharge Support Teams
- Supported Discharge Service Teams
- Multi-Systemic Therapy Teams
- Adolescent Intensive Support Services

Please note these titles reflect only a small, and geographically limited, sample of those which may reflect the practice of ICCS.

The group also discussed models which are not considered to be ICCS. Amongst others, standard community services, liaison teams, assessment teams and others whose work is low frequency were considered to fall outside of the definition. Diagnosis specific teams (such as early-intervention psychosis, learning disability or eating disorder services) could fall under ICCS; however, such services are beyond the scope of this paper.

### Organisational boundaries

Beyond the overarching definition, the expert group discussed and agreed factors relating to the appropriate or ideal organisational boundaries of ICCS teams. The first point discussed related to admission and intake procedures. Like many services, ICCS teams should serve a pre-determined population and use measurable and operationally defined criteria to screen out referrals that do not meet the intake criteria for the service. The group agreed that CYPs admitted to ICCS should comply with the explicit admission criteria (such as age, severity of need, etc.) pre-determined by the service and the team should aim to be resilient against organisational pressure, such as waiting lists, to admit CYPs who do not meet the pre-agreed criteria. The responsibility and scope of ICCS teams was also discussed by the expert group; alongside case management, ICCS should seek to take a broad psychosocial approach to addressing the varied needs of the CYPFs they support. Areas that ICCS teams support with, may include, but are not limited to, the provision of psychiatric services, psychological therapies, housing support, educational support, substance related treatment, social care and safeguarding, employment and rehabilitative services. Formulations and care plans which support CYPS to have agency in their treatment should also be in place; these should include evidence-based interventions and incorporate a systemic approach.

The appropriate provision of after-hours support was also discussed. It was agreed that ICCS should provide, at least, some provision beyond Monday to Friday 9am–5pm; such provision ensures that ICCS are able to respond to and care for psychiatric crises as and when they arise. Although ICCS are predominately community-based treatment models, the responsibility of teams in relation to potential hospitalisation was discussed. The expert group determined that, whilst it might be a long-term goal for ICCS to lead on decisions to admit CYPs who require inpatient treatment, it may be enough, at this stage in ICCS’ development, to aim for involvement in such decisions in collaboration with CYPFs. The importance of maintaining a close relationship between ICCS and inpatient services was confirmed by the ex-service user involved in this study. Discharge procedures from ICCS teams were also discussed and, in particular, the nature of ICCS as a time-limited goal-oriented intervention was noted. ICCS should regularly review the needs of CYPFs and check the eligibility of CYPFs for their service. The group determined that ICCS should aim to form a treatment pathway. The goal, to *safely* return patients to regular community clinic care in the shortest time possible, should be a key focus of care planning reviews and subsequent discharge decisions.

### Human resources

The group determined that, in order to deliver the treatment model detailed above, a range of staffing and personnel issues related to ICCS teams and co-ordinating institutions must also be considered. The number of staff, the roles required, caseload and structure were amongst the areas that were debated and agreed by the expert group. The caseload per clinician is a critical factor in determining the capacity and quality of care that can be provided by each staff member in an ICCS team. The maximum caseload agreed by the group was 5 clients per full-time equivalent (FTE). Although this figure was agreed, the group recognised that great variation currently exists with regards to caseload in ICCS. As such, and if a caseload of 5 clients or fewer per FTE is inappropriate or unattainable, ICCS should ensure that caseloads are small enough to allow for intensive community work and which proffer the flexibility required in order to meet with CYPFs when, and how frequently, they need to be seen. This flexibility was, in particular, supported by the ex-service user involved in this project; they reported that flexibility to meet when and where required was key to their successful involvement with ICCS. Alongside caseload, the minimum clinician contact for each CYPF was also discussed and agreed upon by the group. It was determined that in order to qualify as ICCS, 90% or more of the CYPFs on caseload should have a minimum of two episodes of direct clinical contact with at least one clinician each week. Regular contact was deemed important by the young person involved in this paper; they noted that regularity enabled them to build trusting relationships faster which, ultimately, supported their treatment. The type of contact offered by ICCS teams may vary (face-to-face versus telephone versus video call) dependent on the need and preference of the CYPF. In addition to supporting the CYPFs on the caseload, on at least 1 day per week, the ICCS team should meet and discuss each CYPF on the caseload; the discussion may be brief or detailed depending on each CYPF’s immediate needs or crisis status.

The following points relate to the staffing structure of ICCS teams and the minimum capacity with which they can operate as agreed by the expert group. The first point relates to the role and practice of the ICCS lead; whilst the lead may not have a caseload themselves, they should maintain connection to, and knowledge of, the caseload as a whole. The expert group also determined that ICCS teams should, as a minimum, have access to MDT resources including to one professional who is legally able to provide diagnoses, prescribe medication and who is also able to apply mental health legislation (such as the Mental Health Act, 1983 as utilised in England and Wales). The importance of regular clinical supervision for ICCS team members was also considered by the group. As a minimum, ICCS should aim to provide individual clinical supervision at least once per month. A separate space that provides reflective practice for the whole team is also recommended. The final point of discussion with regards to human resources related to the minimum size of ICCS teams. In order to consistently provide the necessary staffing diversity and coverage required in the ICCS model, the minimum size of a team should be at least four FTE staff members.

### Nature and scope of services

The group engaged in detailed conversation about the nature and scope of services that should be provided by ICCS teams described above. A primary point of discussion related to the location of ICCS interventions. As community-based services which seek to support CYPs to cope with community re-integration following psychiatric emergency, it was agreed that a minimum of 80% of face-to-face contacts for ICCS teams should take place in the community. It was also agreed that ICCS team members should proactively promote a range of community resources, such as sport and activity clubs, to CYPFs. Beyond promotion of such services, ICCS team members should also directly support CYPS to engage in these. Support may include, but is not limited to, travelling with CYPs, attending early sessions or supporting with the booking or organisation of such activities. The second point of discussion related to levels of engagement required from CYPs on the caseload. Primarily, ICCS teams should take a considered, flexible and persistent approach to attempting to engage with CYPs; however, CYP’s consent is critical in such an approach. The need for flexibility around appointments was confirmed by the ex-service user involved in this project; they stated that in ICCS, “I wasn’t given up on or made to feel guilty, [instead,] we would come up with different ways to move forward and [to] stop me from withdrawing.” Alongside the amount of engagement, the methods of engagement with CYPs on the caseload were also discussed. ICCS teams should utilise a range of mechanisms for engagement with CYPs, and they should have a well-considered engagement strategy in place to enable this. The final points of discussion related to the frequency and intensity of face-to-face contact that should be provided by ICCS teams. As an intensive treatment model, it was agreed that at least 2 h of direct clinical contact should be provided to each CYPF on the caseload per week. The rate of contact with CYPs families or informal support systems was also considered; as ICCS should seek to work holistically, engagement with CYPs network (including family, teachers, community leaders) is encouraged. In response, the expert group agreed that ICCS should meet with the wider network of CYPs, with or without the CYP present, on a regular basis; an example of such holistic practice is the Care Programme Approach [37].

### Evaluation and outcome

The importance of robust evaluation and monitoring within ICCS was also discussed by the expert group and confirmed by the young person involved in this process. ICCS should consistently monitor CYPs progress using a variety of pre-determined outcome measures related to mental health and psychological wellbeing such as the Children’s Global Assessment Scale (CGAS) [38] or the Health of the Nation Outcome Scales for Children and Adolescents (HoNOSCA) [39]. Utilising these outcome measures does not only support ICCS teams to review and monitor their service outcomes but, according to the young person involved in this paper, CYPs benefit from the reflection and ‘check-in’ that these measures offer. Alongside formal measures, ICCS should also complete regular service evaluation and should seek feedback from community teams and stakeholders in order to measure and maintain a high quality of care and service.

## Discussion

This paper, and the discussion to which it pertains, has sought to provide clarity and consensus regarding the nature and scope of ICCS. Alongside determining the key features of ICCS (a primarily community-based, high-intensity crisis service), consensus was reached amongst the group in relation to four key areas: organisational boundaries, human resources, nature of services and evaluation and outcome. Key findings were that ICCS should provide at least some out-of-hours support and a minimum of 90% CYPs should receive direct clinical contact at least twice per week. The maximum caseload should be approximately 5 clients per FTE, and the minimum number of staff required to effectively facilitate ICCS is 4 FTE. The importance of supporting CYPs connection with their community and of regular meetings with CYPs wider family and informal support network were also found. Finally, the need for regular and robust evaluation for ICCS was confirmed.

Whilst this paper sets out the minimum requirements of ICCS, the expert group also discussed the future aims of ICCS and what ‘best practice’ may come to entail. Access to education was a key focus of this discussion. ICCS should, at this stage, support CYPs to safely return to school following crisis; however, in the future, the routine provision of day schooling in the ICCS model may bridge the gap between crisis and return. The critical importance of creating and maintaining CYPs connection to their communities is highlighted throughout this paper. In future, ICCS may wish to develop this further and employ a model wherein mental health treatment methods take the form of social and community activities. Finally, the inclusion of support workers in the staffing composition of ICCS should also be considered. Support workers are often young and are anecdotally very good at engaging with CYPs in mental health crisis. In addition, they are a low-cost resource and the value they may bring to the ICCS process should be further explored.

Although the minimum requirements for ICCS were agreed upon by the expert group, an enduring theme of discussion was the sheer variety of models that ICCS may entail. Alongside varied levels of resources and organisational structures, differences in relation to the geography of ICCS were also discussed. The inner-city model of an ICCS, for example, is likely to vary significantly from that of a rural one and such variety should be considered when designing and delivering ICCS. On a practical level, services which offer intensive community-based care may differ in their delivery; however, the shared philosophy of ICCS, of seeking to co-create mental health care alongside CYPFs and stakeholders, is, and should always remain, a key commonality of the practice.

### Strengths and limitations

To our knowledge, this paper was the first of its kind in applying a consensus seeking model to ICCS. As such, this paper provides novel guidelines which can be used to develop services to effectively support CYPFs in crisis. The variety of professionals involved in the production of this paper is a particular strength. A wide variety of multi-disciplinary professionals including psychiatrists, psychologists, nurses and allied health professionals were involved in the process. In addition, and perhaps an even greater strength, was the inclusion of a young person (now 18+) who had previously been a service user of ICCS; their expertise via experience was of great help and their perspective on guidelines was warmly welcomed. Another strength of this paper was the range of countries represented in the consensus process; professionals from Austria, Germany, Spain, France and the UK were involved. Notwithstanding this diversity, many countries were not represented in the process despite great efforts otherwise, the paper remains UK-centric. Similarly, as participants in the meeting were largely identified via pre-existing research and clinical collaborations, leading ICCS clinicians and researchers from around the world may have not been involved; we look forward to working widely and collaboratively in future. This paper also did not have the capacity to explore culturally specific elements of ICCS, and future work in this area should do so. Due to its capacity, this work did not explore aetiologic or systemic factors of mental illness and mental health crisis in young people; researchers may wish to consider these in future development of ICCS models. A final limitation relates to the methodology employed in this paper; whilst a free-flowing and open group discussion was facilitated to gain consensus on the points discussed, a more structured model, such as the Delphi method, may have provided increased methodological robustness. Despite the potential benefit, such methods seek to reach consensus with group members operating in isolation. The varied clinical experiences of group members, and the ranging scope of ICCS across the UK and more widely, meant that active, live and collaborative group discussion was deemed necessary for the creation of these guidelines.

## Conclusions

This paper, which is the product of a round-table meeting of experts, has defined the minimum requirements of ICCS. Despite noting great variety in the current provision of ICCS, areas of agreement, as detailed throughout this paper relate to organisational boundaries, staffing concerns, the scope of an appropriate treatment model and to the evaluation of ICCS. It is hoped that this paper may now come to serve as the basis of clinical guidelines and associated policy documents both in the UK and around the world. In addition, by using this paper, clinicians, academics and related colleagues working in ICCS may seek to further develop the evidence base for this treatment model. Not only should the efficacy of ICCS be further explored in robust and methodologically sound trials, the comparison of ICCS to inpatient and non-intensive community services should also be investigated.

## Data Availability

Not applicable.

## Abbreviations

CYP: Children and young people
NHS: National Health Service
CYPF: Children, young people and their families
ICCS: Intensive community care services
NICE: National Institute for Health and Care Excellence
FTE: Full time equivalent
CGAS: Children’s Global Assessment Scale
HoNOSCA: Health of the Nation Outcome Scales for Children and Adolescents
